# Progress of HOTAIR-microRNA in hepatocellular carcinoma

**DOI:** 10.1186/s13053-022-00210-8

**Published:** 2022-01-29

**Authors:** Bing-rong Wang, Dong-xia Chu, Mei-yu Cheng, Yu Jin, Hao-ge Luo, Na Li

**Affiliations:** 1 Department of Pathophysiology, College of Basic Medical Sciences, Jilin University, 126 Xinmin Street, Changchun, Jilin, 130012 People’s Republic of China; 2 The Basic Medical College, Jilin Medical University, Jilin, 132013 China

**Keywords:** Hepatocellular carcinoma, HOTAIR, MicroRNA, PRC2, ceRNA

## Abstract

The Hox transcript antisense intergenic RNA (HOTAIR) has been identified as a tumor gene, and its expression in HCC is significantly increased. HOTAIR is associated with the proliferation, invasion, metastasis and poor prognosis of HCC. In addition, HOTAIR can also regulate the expression and function of microRNA by recruiting the polycomb repressive complex 2 (PRC2) and competitive adsorption, thus promoting the occurrence and development of HCC. In this review, we discussed the two mechanisms of HOTAIR regulating miRNA through direct binding miRNA and indirect regulation, and emphasized the role of HOTAIR in HCC through miRNA, explained the regulatory pathway of HOTAIR-miRNA-mRNA and introduced the role of this pathway in HCC proliferation, drug resistance, invasion and metastasis.

## Introduction

Hepatocellular carcinoma (HCC), as the fifth most common human malignant tumor, is the second leading cause of cancer-related death in the world [1, 2]. HCC accounts for 90% of primary liver cancer cases, with high malignancy and poor prognosis [3]. At present, the potential molecular mechanism of HCC has not been discovered, so surgery, radiotherapy and chemotherapy are still the main treatment for HCC [1, 4, 5].

Non-coding RNA has been proved to be involved in the development, progression, invasion, metastasis and drug resistance of cancer. NcRNA can be divided into two categories according to their length: small non-coding RNA (< 200 nt) and long non-coding RNA (lncRNA >200 nt) [6]. LncRNA was originally considered as a transcriptional noise. Studies have found that lncRNA plays an indispensable role in tumor cell growth, drug resistance and tumor stem cell formation [6, 7]. HOTAIR, as a kind of lncRNA, is a 2158 bp RNA and located on chromosome 12 [6, 8]. HOTAIR is the first lncRNA found to have reverse transcription effect, which plays a role in many diseases, such as tumor, rheumatoid arthritis, heart disease and so on [9–11]. HOTAIR has been proved to be highly expressed in many cancers and regarded as a tumor gene [12]. However, the high expression of HOTAIR can promote the recurrence and metastasis of hepatocellular carcinoma, affect the prognosis of patients and shorten the survival time of patients [13]. Therefore, HOTAIR has been regarded as a new tumor marker of HCC [9, 14, 15].

MicroRNA (miRNA) is a kind of endogenous non-coding RNA with 19 ~ 25 nt, which can directly bind to the 3′ untranslated region (3′-UTR) of messenger RNA (mRNA) [16]. MiRNA can regulate gene expression by degrading transcripts or interfering with protein transformation, thus inhibiting protein transformation in various biological and pathological processes [17]. In addition, miRNA has been indicated to play an important role in the progression of cancer [18–20].

**Fig. 1:**
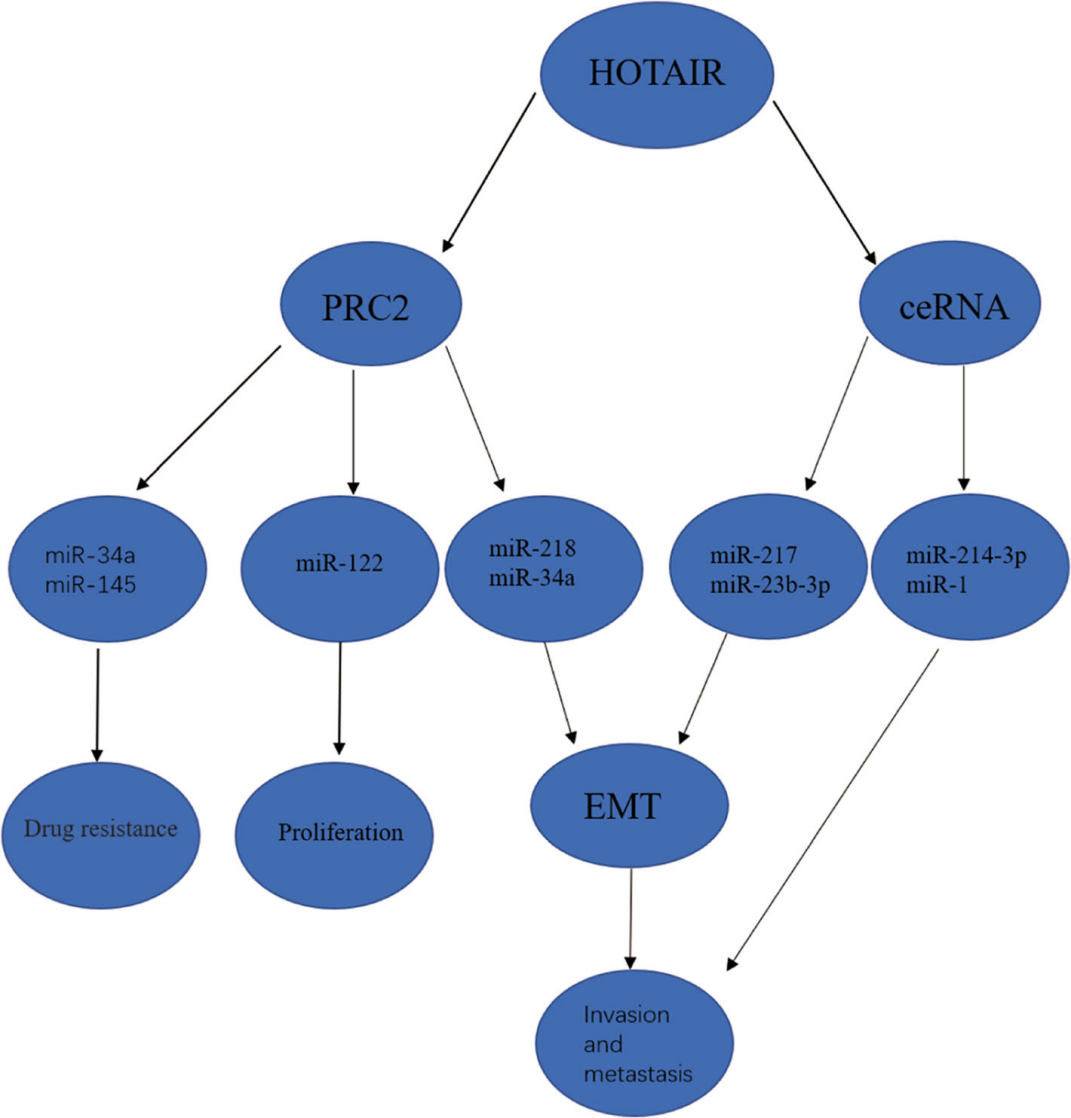
HOTAIR regulates microRNA by binding to PRC2 or competitive adsorption, thereby promoting the proliferation, invasion, metastasis and drug resistance of HCC cells

There are two main ways of HOTAIR regulation of miRNA: direct regulation and indirect regulation. Indirect regulation is that HOTAIR can silence miRNA by binding with PRC2 to form a silencing complex, which targets the promoter region of miRNA. Direct regulation is that HOTAIR, as a competitive adsorption of miRNA, participates in various regulation pathways. By studying the regulation of HOTAIR on miRNA, we can conclude that HOTAIR can be used as a new target for tumor therapy and provide new ideas for tumor therapy (Fig. 1).

### HOTAIR regulate miRNA by binding to multi-comb inhibitory complex 2 (PRC2)

#### The mechanism of the combination of HOTAIR and PRC2

HOTAIR molecules have complex secondary structures and scan perform complex biological functions [21]. Based on the fragment analysis of HOTAIR, it is found that HOTAIR can be identified in four independent domains (D1 ~ D4). Among these four domains, D1 (nucleotide 1–530) consists of 12 helix, 8 terminal loops and 4 junctions. The gel shift assay determined the binding affinity of D1 and PRC2 [22]. Experiments showed that the secondary structural elements of the PRC2 binding fragment were particularly stable and limited to HOTAIR D1 [21, 23]. PRC2, as one of the most important complexes of epigenetic modification gene PcG family, is mainly composed of three subunits: EZH2, SUZ12 and EED. Among them, EZH2 is a highly conserved histone methyltransferase that can target histone H3K27 and is a catalytic subunit of PRC2 molecule [24]. After being combined with SUZ12 and EED, it can modify histones and silence genes [25].

#### HOTAIR promotes the occurrence of HCC by combining with PRC2

**Fig. 2:**
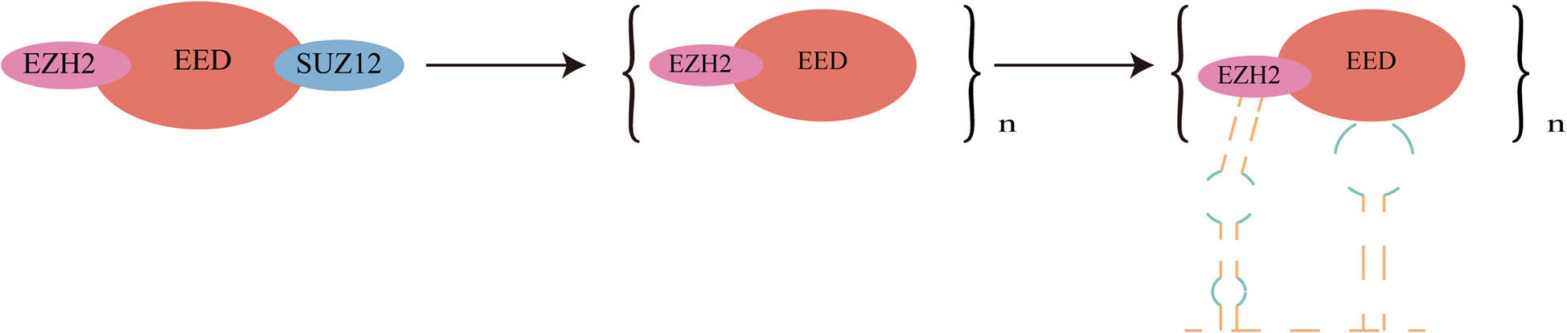
HOTAIR can promote the degradation of SUZ12 and then bind to PRC2 to regulate histone modification and chromatin state

This progress that HOTAIR regulates histone modification and chromatin state occurs in the nucleus. HOTAIR is one of the most studied lncRNA interacting with PRC2, which can be used as a modular scaffold to bind to PRC2 and LSD1 [24]. HOTAIR mainly binds to EZH2 when interacting with PRC2. HOTAIR can inhibit gene transcription through PRC2 trimethylation of histone H3 Lys 27 (H3K27me3). The 3’end of HOTAIR has a 636 nt region that can bind to LSD1/CoREST/REST. LSD1 is a flavonoid-dependent monoamine oxidase, which can form a multi-protein complex that is a key participant in gene silencing. With REST (RE1-silencing transcription factor) and CoREST. Therefore, HOTAIR can play a role in silencing miRNA by binding to PRC2 and LSD1 [25] (Fig. 2).

**Fig. 3:**
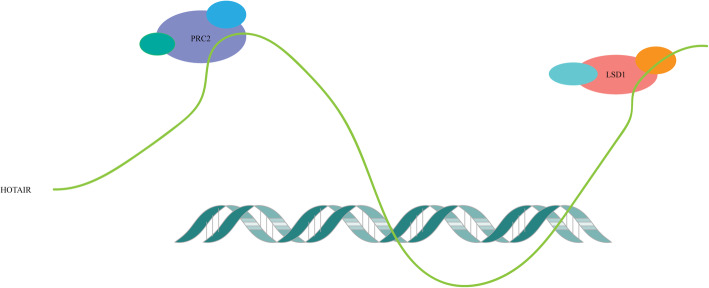
The 3’end of HOTAIR can be combined with LSD1, and the 5’end can be combined with PRC2 to form a complex. Then this complex combines with DNA to form a DNA-RNA trimer to induce gene silencing

Suppressor zeste 12 homolog (SUZ12), as an important binding subunit of PRC2, undergoes proteasome degradation. HOTAIR overexpression can promote this degradation process and enhance the PLK1-dependent ubiquitination of SUZ12. HOTAIR can also be used as a scaffold for PRC2 to connect with DEAD box protein 5 (DDX5) [26]. DDX5 can stable SUZ12, and then stabilize SUZ12 and PRC2-mediated gene silencing for the reason that DDX5 can replace the original Mex-3 RNA binding family member B (Mex3b) linked to HOTAIR. Patients with hepatocellular carcinoma whose HOTAIR and PLK1 expression increased more than twice, the expression of PRC2 target gene and EPCAM also increased significantly [26, 27] (Fig. 3).

### HOTAIR regulates miRNA expression by binding to PRC2

After recruiting PRC2, HOTAIR can target the miRNA promoter region, making the miRNA promoter region rich in H3K27me3 modification, and then silencing the expression of miRNA. HOTAIR can regulate multiple tumor-related cell pathways through PRC2-regulated miRNA pathways. For example, HOTAIR can inhibit the resistance of gastric cancer cells to cisplatin by inhibiting miR-34a to regulate PI3K/Akt and Wnt/−catenin signaling pathways [28]. HOATIR can also affect the cell cycle of HCC and regulate the proliferation, invasion and metastasis of tumor cells.

#### HOTAIR regulates EMT signaling pathway via miRNA

Epithelial-mesenchymal transition (EMT) is a process in which epithelial cancer cells acquire mesenchymal characteristics, is one of the key steps in tumor metastasis, and can also predict the poor prognosis of tumor patients [29]. HOTAIR, as an oncogene, can regulate EMT in various ways and maintain E/M mixed phenotype to promote the metastasis of HCC cells. C-Met is a receptor tyrosine kinase that contributes to the initiation, development and progression of HCC. HOTAIR knockout can inhibit the expression and activation of C-Met protein, thus inhibiting the activation of MAPK and STAT3 that are downstream pathways of C-Met. HOTAIR knockout can also inhibit the invasion and metastasis of HCC cells induced by C-Met [30]. HOTAIR-C-Met axis can act as regulators of epithelial/mesenchymal hybridization of hepatocytes and accelerate the speed-limiting step of tumor metastasis [31, 32].

HOTAIR can also regulate EMT by regulating a variety of miRNAs. For example, the HOTAIR-PRC2 complex can bind to the miR-34a promoter region, inhibit the expression of miR-34a in the nucleus, and then change the HGF/c-Met/Snail signaling pathway to promote EMT [31, 32]. In addition, HOTAIR/miR-613/c-Met axis can regulate the occurrence and development of retinoblastoma cells and EMT process [33]. HOTAIR can also inhibit the transcription of E-cadherin, an EMT marker, by regulating the activity and localization of Snail2.

MiR-218, as a tumor inhibitor, can inhibit cell migration/invasion and regulate multiple signal pathways in HCC cells. For example, miR-218 can reverse epithelial-end body transition (EMT) to mesothelial-epithelial transition (MET); MiR218 may also regulate the P14 and P16 signaling pathways [34]. HOTAIR can target miR-218 promoter region by binding with EZH2, negatively regulate miR-218, and then regulate its downstream signal pathway [35].

#### HOTAIR regulates the proliferation of hepatocellular carcinoma via miRNA

HOTAIR can promote the proliferation of various tumor cells by regulating miRNA. For example, HOTAIR can target miR-126 to activate CXCR4 and RhoA signaling pathways and promote the proliferation of gastric cancer cells [36]. HOTAIR can also promote the proliferation, migration, invasion and apoptosis of breast cancer cells by regulating miR-20a-5p/HMGA2 axis [37].

MiR-122 is the main liver-specific miRNA, which can regulate the invasion and metastasis of liver cancer [38]. HOTAIR can upregulate the expression of DNA methyltransferase DNMTs by binding with EZH2, thus leading to DNA methylation mediated by DNMTs in HCC cells and epigenetically inhibits the expression of miR-122. Cyclin G1 (CCNG1) is the functional target of miR-122. In hepatoma cells, miR-122 can promote the proliferation of HepG2 cells through G1-p53 axis. Studies have shown that after HOTAIR knockout or miR-122 ectopic expression, CCNG1 expression increased, the percentage of HCC cells in G1 phase increased, while the number of cells in S phase decreased [39]. However, the overexpression of miR-122 can reverse the cell proliferation induced by pHOTAIR [38, 40]. It can be seen that HOTAIR can inhibit the expression of miR-122, activate CCNG1 and increase the promotion of tumorigenicity in HCC by HOTAIR/miR-122/Cyclin G1 pathway.

#### HOTAIR regulates drug resistance of HCC through miRNA

Traditional radiotherapy and chemotherapy are still the main treatment methods for HCC, while traditional anti-tumor drugs such as paclitaxel and cisplatin greatly reduce the treatment efficiency of chemotherapy due to their drug resistance [41, 42]. HOTAIR has now been confirmed to be related to drug resistance of HCC cells. For example, the STAT3/ABCB1 signaling pathway can be inhibited after HOTAIR knockout, and the sensitivity of HCC cells to cisplatin increases [43].

HOTAIR can also influence the drug resistance of HCC cells to anti-tumor drugs by regulating miRNA. For example, miR-34a, as the downstream target of tumor suppressor gene p53, has been proved to have the effect of inhibiting tumor occurrence and development. Studies have shown that HOTAIR can down-regulate miR-34a to promote the occurrence and development of tumors in breast cancer, gastric cancer and other tumors [28]. HOTAIR regulates miR-34a by forming EZH2-HOTAIR complex, which specifically binds to the promoter region of miR-34a to silence miR-34a, and then regulates multiple signal pathways such as EMT, PI3K/Akt and Wnt/−catenin [28, 44].

Paclitaxel is a commonly used chemotherapy drug for HCC. The therapeutic effect of paclitaxel is weakened due to the enhancement of drug resistance of HCC. However, studies have found that Akt kinase signaling pathway can induce drug resistance of hepatocellular carcinoma through PI3 kinase or Akt itself. HOTAIR can down-regulate miR-34a in HCC to activate Akt phosphorylation and Wnt/−catenin signaling pathway, which proves that HOTAIR can regulate the resistance of HCC to paclitaxel in this way [45]. These studies also prove that HOTAIR can be used as a marker of paclitaxel resistance of HCC, and provide a new molecular target for improving the efficiency of chemotherapy treatment of HCC.

Multidrug resistance (MDR) is very common in tumor patients and is one of the main reasons leading to the low efficiency of chemotherapy. MDR is related to the transformation and overexpression of growth factor + 1 (TGF-β1), drug outflow transport agent P-glycoprotein (P - gp) and breast cancer drug resistance protein. TGF-β1, as a multifunctional cytokine, can regulate the growth, apoptosis and differentiation of tumor cells and promote EMT invasion and metastasis of tumor cells. TGF-β1 can increase HOTAIR expression through SMAD4 in HCC. MiR-145 can directly interact with BCRP and 3′-UTR of P -gp. HOTAIR can inhibit miR-145 expression by binding to PRC2. Therefore, TGF-β1 can inhibit BCRP and P -gp through HOTAIR/PRC2/miR-145 axis, and then regulate multidrug resistance of HCC.

## HOTAIR, as a competitive endogenous RNA (ceRNA), adsorbs miRNA and then regulates the progression of hepatocellular carcinoma

HOTAIR, as ceRNA, can competitively adsorb a variety of miRNAs, thus relieving the inhibition of miRNA on the expression of target mRNA [46]. The imbalance between ceRNA and miRNA plays an important role in the occurrence and development of tumors [47]. HOTAIR-miRNA-mRNA regulatory network regulates the process of HCC through this way. The progress that HOTAIR functions as a ceRNA mainly occurs in the cytoplasm.

### HOTAIR regulates HCC through sponging miR-217 in multiple ways

MiR-217 has been proved to be a tumor suppressor gene, which can play a regulatory role in gastric cancer, renal cancer, lung cancer and other tumors [48–51]. MiR-217, as a direct target of HOTAIR, affects the proliferation, migration, invasion and EMT of HCC. In HCC, when miR-217 is inhibited, p27 expression is down-regulated, while CCND1 expression is up-regulated [52]. HOTAIR can negatively regulate miR-217 to regulate cell proliferation and cell cycle. HOTAIR can also affect the invasion and metastasis of tumor cells by adsorbing miR-217-5p. As an oncogene, HOTAIR can inhibit the cell process of HCC after being silenced. The p-PI3K/p-AKT/MMP-2/9 signaling pathway is closely related to cancer cell migration, invasion and metastasis, and miR-217-5p inhibitors can restore the effect of HOTAIR silencing on HCC cells by activating this pathway [53].

At the same time, HORTAIR can also affect the drug resistance of liver cancer cells to sorafenib through miR-217. Sorafenib, as a multi-target kinase inhibitor, has been approved as the only target drug for the treatment of advanced HCC. However, due to its drug resistance, the therapeutic effect is often limited. Studies have shown that the drug resistance of sorafenib increases in liver cancer cells overexpressing HOTAIR [50]. However, in liver cancer cells with HOTAIR knockout, it was found that the expression of E-cadherin increased while the expression of Vimentin decreased, and the drug resistance of sorafenib decreased. Also, HOTAIR can negatively regulate the expression of miR-217. After HOTAIR knockout, the expression of miR-217 increased, and the sensitivity of sorafenib in the treatment of liver cancer increased. That is, HOTAIR can increase the drug resistance of sorafenib by inhibiting the expression of miR-217 [54]. The results of research can provide new ideas for the treatment of liver cancer.

It can be seen that HOTAIR-miR-217 can affect HCC in many ways and become a new target for treating HCC.

### HOTAIR affects the metabolism of hepatocellular carcinoma cells through competitive adsorption of miRNA

Abnormal metabolism is one of the signs of cancer. According to Waberg effect, tumor cells give priority to glycolysis to supply energy even under the condition of sufficient oxygen. Micro RNA plays an important role in aerobic glycolysis.

MiR-143 plays an important role in regulating glycolysis and tumor cell proliferation [55, 56]. In lung cancer, miR-143 reduces the expression of hexokinase 2 (HK2) protein by targeting the mammalian target of rapamycin (mTOR) pathway. HOTAIR can also regulate HK2 expression in esophageal squamous cell carcinoma by combining endogenous miR-125 and miR-143, and then regulate glycolysis of esophageal squamous cell carcinoma to affect the occurrence and development of tumors [57].

HCC is highly dependent on glycolysis. HOTAIR can promote glycolysis by up-regulating mTOR-induced glucose transporter isoform 1 (GLUT1) and activating mTOR signal transduction [58]. GLUT1 is an important rate-limiting factor for glucose transport and metabolism in cancer cells [59]. The expression of GLUT1 in hepatocellular carcinoma was significantly increased. And there is hypoxia response element (HRE) in HOTAIR promoter region, and HRE can bind to hypoxia inducible factor HIF [60, 61]. HIF1A can be used as a functional target of miR-130a-3p in HCC. HOTAIR can inhibit HIF by competitively adsorbing miR-130a-3p to relieve the repression of miR-130a-3p on HIF1A. Experiments have verified that HOTAIR/miR-130a-3p/HIF1A network can regulate the viability of HCC cells under hypoxia by regulating metabolic reprogramming under hypoxia conditions, and can reduce the viability of HCC cells [62].

OATP 1B1 is an important drug transporter, which plays an important role in the liver absorption of therapeutic drugs and can affect the elimination rate of drugs in the liver [63]. Studies have found that HOTAIR can “sponge” miR-206/miR-613 in liver cancer cells, thus destroying the binding site of miR-206/miR-613 and OATP1B1 mRNA 3’-UTR, affecting the binding of miR-206/miR-613 and OATP1B1 mRNA 3′-UTR, and then eliminating the stimulation of LncRNA HOTAIR on OATP1B1 protein. Thus, HOTAIR can affect the liver’s ability to metabolize tumor drugs by affecting the binding of miR-206/miR-613 to OATP1B1 [64].

### HOTAIR regulates miRNA as ceRNA to promote invasion and metastasis of HCC

HOTAIR is an oncogene. In HCC, overexpression of HOTAIR can inhibit the expression of adhesion-related integrin, delay the adhesion of HCC cells, and obtain higher metastasis ability [65–67]. The mechanism of HOTAIR promoting invasion and metastasis of HCC cells is complex, but studies have shown that HOTAIR can competitively adsorb a variety of miRNA to further regulate downstream targets, thus promoting invasion and metastasis of HCC. FOXC1 is a marker of cancer and related to poor prognosis of HCC [68]. HOTAIR can bind to the promoter region of FOXC1, the expression of FOXC1 is increased, and the transcription level of HOTAIR is increased. HOTAIR can also adsorb miR-1, down-regulate the expression of miR-1 to promote the invasion and metastasis of HCC, and form FOXC1/HOTAIR/miR-1 network to regulate the invasion and metastasis of HCC [69, 70].

As a tumor suppressor, miR-214-3p is obviously low expressed in HCC, glioma and other tumor cells. HOTAIR can regulate Flotillin 1 (FLOT1) by competing with endogenous miR-214-3p. FLOT1 is a key tumor gene, which can be used as a stable scaffold when recruiting multi-protein complexes, and is related to the invasion and metastasis of tumors [69]. Therefore, HOTAIR/miR-214-3p/FLOT1 axis can regulate the invasion and metastasis of HCC.

Zinc-finger E-box-binding homeobox 1 (ZEB1) is a key factor to promote EMT. It is abnormally expressed in many cancers and can promote the migration, invasion and metastasis of tumor cells [71]. HOTAIR can “sponge” miR-130a-5p to regulate ZEB1 and thus promoting malignant progression of esophageal squamous cell carcinoma, while HOTAIR is regulated by chemokine (C-C motif) ligand 18 (CCL18), which plays an important role in tumor progression and metastasis [71]. In HCC, HOTAIR can directly bind to miR-23b-3p to down-regulate miR-23b-3p, and miR-23b-3p can negatively regulate ZEB1, and then participate in the regulation of EMT [72]. Therefore, we can also predict that CCL18 can regulate the occurrence and development of HCC through HOTAIR-miRNA-ZEB1 axis.

## The relationship between HOTAIR and tumor genetic susceptibility

The occurrence and development of HCC is a complex process, which is related to gene changes and living environment [73]. Epidemiological studies have shown that hepatitis B virus (HBV) or hepatitis C virus (HCV) infection and drinking are the main pathogenic factors of HCC, but only a part of people will suffer from HCC, which means that the susceptibility of HCC is related to genetic factors [74]. Studies have shown that HOTAIR, as a kind of LncRNA, can affect the occurrence and development of HCC by interrupting gene expression at genetic and epigenetic levels [75].

Single nucleotide polymorphism (SNP) refers to the gene polymorphism caused by single nucleotide mutation in human genome, which is one of the common genetic changes in human genome [76]. With the deepening of research, it has been found that SNP can appear in both coding and non-coding regions of genes, and SNP can also affect the expression and function of LncRNA, which is related to the susceptibility of diseases to cancer [77, 78]. For example, HOTAIR SNP rs920778 is significantly associated with lung cancer susceptibility which is a protective factor in female patients and non-smokers [79]; HOTAIR rs12826786 SNP increases the incidence of cardiac adenocarcinoma in North China [80]; The rs920778 and rs12826786 polymorphism of HOTAIR increased the incidence of breast cancer in women in southeastern Iran, while rs1899663 had the opposite effect [81]. RS7958904 polymorphism can reduce the risk of colorectal cancer [77]. However, as a gene mutation, the effect of SNP on tumor is limited by factors such as race, region and environmental hazards. For example, the CC genotype of rs920778 polymorphism was significantly associated with breast cancer risk in Turkish population, but no such association was found in Chinese samples [82, 83]. Similarly, the T allele of rs12826786 can increase the risk of gastric cancer and adenocarcinoma in Chinese population [80, 84]. But the same mutation found in the Turkish population is not associated with genetic susceptibility to gastric cancer, which may be due to differences in race and genetic background [78].

The single nucleotide polymorphism of HOTAIR can also affect the susceptibility to HCC, and it is hereditary. Studies have shown that the C allele of HOTAIR rs12427129 can relax the regulation of gene expression by affecting the combination of HOTAIR with protein and downstream miRNA and genes, which is helpful to the gene susceptibility of HCC. However, rs3816153 located in the upstream region of HOTAIR can affect hypermethylation of promoter and enhancer in liver tissue. After mutation, the risk of GT/TT genotype is 30% higher than that of GG genotype, which promotes the occurrence and development of HCC. Therefore HOTAIR rs12427129 and rs3816153 are significant genetic susceptibility factors for HCC. In addition, rs920778 may be the regulatory SNP of HOTAIR, which can regulate the expression of HOTAIR. Compared with CC genotype, the TT genotype of HOTAIR contributes to the genetic susceptibility and proliferation of HCC [85, 86].

These studies suggest that the single nucleotide polymorphism of HOTAIR is related to the genetic susceptibility to HCC, but more investigations and studies are needed to prove the specific role.

## Discussion and future perspectives

At present, liver cancer, as a malignant tumor disease with low detection rate, high malignant degree and poor prognosis in the early stage, is still a difficult problem in the field of medical research [1, 2]. However, with the deepening of researches on non-coding RNA, the expression and regulation mechanisms of various non-coding RNAs have been clarified gradually. HOTAIR, as an important part of lncRNA, has been clearly found to promote the proliferation, invasion and metastasis of tumor cells.

HOTAIR can promote the occurrence and development of HCC in many ways. For example, HOTAIR can activate autophagy and promote the proliferation of HCC cells by up-regulating the expression of autophagy-related 3 (ATG3) and ATG7 expression [87]. HOTAIR can regulate cell cycle by enhancing the activity of STAT3 and down-regulating the expression of cyclin D1 (CCND1), which plays a key role in the proliferation of hepatocellular carcinoma [39]. HOTAIR can down-regulate the expression of CCND1 and CCND2 in ovarian cancer through sponging miR-206, and affect the invasion and metastasis of ovarian cancer cells [88]. At the same time, the expression of HOTAIR was obviously high in HCC, while the expression of miR-206 was obviously reduced. Therefore, we can speculate that HOTAIR can regulate the expression of CCND1 by regulating miR-206 thereby affecting the proliferation of HCC.

HOTAIR can also up-regulate RNA binding motif protein 38 (RBM38), a tumor suppressor, and then promote the invasion and metastasis of HCC [89]. HOTAIR can down-regulate the expression of miR-326 in lung cancer cells, and then increase the expression of SP1, and regulate the cisplatin resistance of lung cancer cells through miR-326/SP1 pathway [90]. At the same time, HOTAIR can regulate chemotherapy (C-C motif) ligand 2 in HCC and promote the proliferation of macrophages and bone marrow-derived inhibitory cells in HCC [91]. Therefore, we assumed that HOTAIR can regulate the expression of CCL2 through miR-326/SP1 pathway, thus promoting the proliferation of macrophages/MDSC and regulating the tumor microenvironment of HCC.

At the same time, miRNA, as a highly conserved tissue-specific small non-protein coding RNA, can maintain cell balance through negative regulation. MiRNA has been proved to be related to many diseases such as heart disease and tumor. Many miRNAs have been identified as tumor inhibitors, which are involved in regulating the occurrence and development of tumors. HOTAIR can bind to a variety of miRNA, participate in many cell signal transduction pathways, and then act on mRNA. Studies have shown that HOTAIR exosomes are related to HER2, and HOTAIR can regulate the expression of HER2 through “sponge” miR-331-3p. Therefore, HOTAIR may also regulate exosomes through miRNA, and then regulate the occurrence and development of tumors [92, 93]. These research results make people realize more possibilities of HOTAIR as a new target for liver cancer. In addition, studies have analyzed and verified the miRNA-mRNA regulatory network of HBV-related HCC, and identified some miRNA-mRNA regulatory pathways that are significant for HCC, such as miR-93-5p/miR-106-5p/miR-21-5p-STAT3, miR-21-5p-PIK3R1 and Jean 7c-5p-NRAS axis [94]. Therefore, miRNA-mRNA has significance in the regulation of HCC. HOTAIR is a known LncRNA that can regulate HCC. The regulation mechanism of HOTAIR-miRNA-mRNA may provide a new target and idea for the treatment of HCC.

## Data Availability

Not applicable.

## Abbreviations

HCC: hepatocellular carcinoma
HOTAIR: Hox transcript antisense
RNA: microRNA
EZH2: Enhancer of zeste homolog 2
PRC2: polycomb-group proteins 2
ceRNA: proliferation; drug resistance; invasion; metastasis; metabolism
